# Exoskeleton-guided passive movement elicits standardized EEG patterns for generalizable BCIs in stroke rehabilitation

**DOI:** 10.1186/s12984-025-01627-7

**Published:** 2025-04-26

**Authors:** Xinyi Zhang, Lanfang Xie, Wanting Liu, Shaoying Liang, Liyao Huang, Mingjun Wang, Lingling Tian, Li Zhang, Zhen Liang, Hai Li, Gan Huang

**Affiliations:** 1 School of Biomedical Engineering, Health Science Center, Shenzhen University, Shenzhen, 518060 Guangdong China; 2 Guangdong Provincial Key Laboratory of Biomedical Measurements and Ultrasound Imaging, Shenzhen, 518060 Guangdong China; 3 Department of Rehabilitation Medicine, Shenzhen Hospital, Southern Medical University, Shenzhen, 518101 Guangdong China; 4 Department of Occupational Therapy, School of Rehabilitation Medicine, Southern Medical University, Guangzhou, 510515 Guangdong China

**Keywords:** Brain computer interface, Electroencephalography, Exoskeleton, Stroke rehabilitation, Generalization ability

## Abstract

**Background:**

Brain-computer interfaces (BCIs) hold significant potential for post-stroke motor recovery, yet active movement-based BCIs face limitations in generalization due to inter-subject variability. This study investigates passive movement-based BCIs, driven by exoskeleton-guided rehabilitation, to address these challenges by evaluating electroencephalogram (EEG) responses and algorithmic generalization in both healthy subjects and stroke patients.

**Methods:**

EEG signals were recorded from 20 healthy subjects and 10 stroke patients during voluntary and passive hand movements. Time and time-frequency domain analyses were performed to examine the event-related potential (ERP), event-related desynchronization (ERD), and synchronization (ERS) patterns. The performance of two BCI algorithms, Common Spatial Patterns (CSP) and EEGNet, was evaluated in both within-subject and cross-subject decoding tasks.

**Results:**

Time-domain and time-frequency analyses revealed that passive movements elicited stronger, more consistent ERPs in healthy subjects, particularly in bilateral motor cortices (contralateral: $-7.29\pm 4.51$ μV; ipsilateral: $-4.33\pm 3.69$ μV). Stroke patients exhibited impaired mu/beta ERD/ERS in the affected hemisphere during voluntary movements but demonstrated EEG patterns during passive movements resembling those of healthy subjects. Machine learning evaluation highlighted EEGNet’s superior performance, achieving 84.19% accuracy in classifying affected vs. unaffected movements in patients, surpassing healthy subject left-right discrimination (58.38%). Cross-subject decoding further validated passive movement efficacy, with EEGNet attaining 86.00% (healthy) and 72.63% (stroke) accuracy, outperforming traditional CSP methods.

**Conclusions:**

These findings underscore that passive movement elicits consistent neural responses, thereby enhancing the generalizability of decoding algorithms for stroke patients. By integrating exoskeleton-evoked proprioceptive feedback, this paradigm reduces inter-subject variability and improves clinical feasibility. Future work should explore the application of exoskeletons in the combination of active and passive movement for stroke rehabilitation.

**Supplementary Information:**

The online version contains supplementary material available at 10.1186/s12984-025-01627-7.

## Introduction

Stroke, a leading cause of long-term disability worldwide, affects approximately 13 million people annually, resulting in 6.4 million deaths and 5 million permanent disabilities [1, 2]. Survivors often face motor, sensory, cognitive, and language impairments, with motor deficits occurring in 80% of cases [3]. While conventional rehabilitation therapies (e.g., physical and occupational therapy) partially restore motor function, they demand substantial human resources and sustained patient adherence [4]. Post-discharge challenges in maintaining high-intensity training further limit long-term recovery [5].

Motor imagery-based brain-computer interfaces (MI-BCIs) integrated with robotic exoskeletons offer a promising solution to enhance rehabilitation outcomes. Exoskeletons provide assistive forces to paralyzed limbs, enabling patients to execute predefined movement patterns [6, 7]. By coupling MI-BCI with exoskeleton control, patients actively engage in brain-driven movements while receiving real-time somatosensory feedback, potentially strengthening corticomuscular connections to promote functional recovery [8]. Clinical trials demonstrate the efficacy of this approach: Ramos-Murguialday et al. reported significant upper-limb motor improvements in chronic stroke patients using MI-BCI-controlled exoskeletons compared to sham feedback [9], while Ono et al. observed similar benefits in severe hemiparesis [4].

Despite these advances, MI-BCI adoption remains limited by training complexity. Effective classifier development requires patients to generate distinguishable EEG signals through prolonged, focused motor imagery (MI) tasks-a process often perceived as monotonous and demotivating [10]. Stroke-related challenges, including limb weakness, cognitive deficits, and attention lapses, further hinder sustained participation [11–13]. Consequently, training duration and quality are constrained by patients’ physical and cognitive limitations [14].

A critical yet underexplored barrier to widespread clinical adoption is the substantial inter-subject variability in EEG patterns during active motor imagery, which severely limits algorithm generalizability across patients. While transfer learning strategies using deep neural networks and adaptive frameworks show promise in healthy subjects [15–17], their application to stroke patients remains challenging [18, 19]. Stroke-induced factors-such as brain lesions, medication effects, and dynamic EEG signal variability-compromise signal quality and system generalizability [20, 21]. Current solutions primarily focus on algorithmic improvements, but addressing variability at the signal source level represents a potentially transformative complementary approach.

Motor pattern heterogeneity further compounds these challenges. Healthy individuals exhibit divergent MI strategies (e.g., movement patterns and temporal features), while stroke patients develop compensatory mechanisms (e.g., mirror or synergistic movements), both of which alter EEG time-frequency features and spatial distributions, exacerbating decoding variability. We propose that exoskeleton-guided standardization of spatiotemporal movement patterns could theoretically reduce this heterogeneity, not as a replacement for active MI paradigms but as a complementary approach to scaffold training and enhance consistency.

However, fundamental gaps persist in our understanding: (1) Neural response gap: Neural distinctions between voluntary movement and (exoskeleton-guide passive movements remain uncharacterized; (2) Pathological signature gap: Post-stroke compensatory effects, interhemispheric activation asymmetry, and their EEG correlates lack systematic investigation; (3) Algorithmic generalizability gap: The impact of movement standardization on machine learning robustness in cross-subject/population scenarios remains unvalidated. To address these gaps, this study integrates neurophysiological mechanism decoding and algorithmic adaptability optimization to investigate three core questions:

1. Voluntary vs. passive movement EEG divergence: How do signal characteristics (e.g., event-related desynchronization/synchronization, or slow-wave potentials) and amplitudes differ between active MI and exoskeleton-driven movements, and what do these differences reveal about motor control and sensory feedback mechanisms?
2. Healthy vs. stroke EEG signatures: What are the distinct patterns of brain activation intensity, spatial distribution, and interhemispheric asymmetry in stroke patients, and how do these reflect functional reorganization post-injury?
3. Algorithmic generalizability: Can movement pattern standardization through exoskeleton assistance enhance cross-subject decoding performance compared to conventional voluntary movement paradigms?

By examining these questions, In this work, we aim to determine whether exoskeleton-guided passive movements, with the support of advanced deep learning algorithms, can exhibit improved cross-subject generalization performance in stroke patients. This approach could potentially address a critical limitation in current BCI-based stroke rehabilitation strategies by reducing neural response variability while preserving therapeutic engagement.

## Methods

### Experiment design

#### Participant

In the study, 20 healthy subjects (HS) and 10 stroke patients (SP) were recruited to participate in the movement related experiments. This study was approved by the Ethics Committee of Southern Medical University’s Shenzhen Hospital (Ethics approval number: NYSZYYEC20220003).

The healthy control group consisted of 20 participants (4 females and 16 males), with a mean age of 25 years. None of the participants had a history of neurological or psychiatric disorders, and all maintained good health throughout the experimental period. Prior to participation, all subjects were fully informed about the tasks involved and signed informed consent forms.

The stroke patient group, as illustrated in Table 1, included 10 individuals (4 females and 6 males) with an average age of 55 years. The conditions of the patients included 7 cases of cerebral infarction and 3 cases of cerebral hemorrhage. Inclusion criteria were as follows: (1) patients diagnosed with their first stroke by a neurologist and confirmed by CT (Computed tomography) or MRI (Magnetic resonance imaging), with the onset occurring at least 1 month prior; (2) individuals with unilateral hemiparesis; (3) upper limb Brunnstrom stages of at least II; (4) right-handed prior to the stroke as confirmed by the Edinburgh Handedness Inventory [22]; (5) adequate cognitive function to follow instructions and complete the study, with MMSE scores within the normal range; (6) capability to withstand the EEG lab environment and complete the required tests. Exclusion criteria included: (1) unstable health condition or serious complications such as congestive heart failure, deep vein thrombosis, severe hypertension, or respiratory or renal impairment; (2) significant cognitive impairments preventing understanding or execution of the tasks; (3) upper limb muscular or skeletal disorders affecting task performance; (4) history of psychiatric disorders or current use of antipsychotic medication; (5) presence of syncope syndrome; (6) severe neglect syndrome. Demographic, clinical, and neuropsychological characteristics of all patients are presented in Table 1. Both stroke patients and control participants were briefed about the research protocol and provided with written informed consent forms before the commencement of the study. In cases where right-sided hemiparesis prevented the participant from signing, a direct relative signed the consent form on behalf of the patient.

**Table 1:** Demographic, clinical, and neuropsychological characteristics of stroke patients

ID	Age	Gender	Type of lesion	Affected side	Months Post-onset
01	74	Female	Cerebral infarction	Left	3
02	58	Male	Cerebral infarction	Right	10
03	44	Male	Cerebral infarction	Right	12
04	52	Male	Cerebral infarction	Left	12
05	35	Female	Cerebral hemorrhage	Right	6
06	54	Male	Cerebral infarction	Right	1
07	75	Female	Cerebral infarction	Right	3
08	63	Female	Cerebral hemorrhage	Left	2
09	36	Male	Cerebral hemorrhage	Right	16
10	59	Male	Cerebral infarction	Left	4

#### Experimental paradigm

**Fig. 1:**
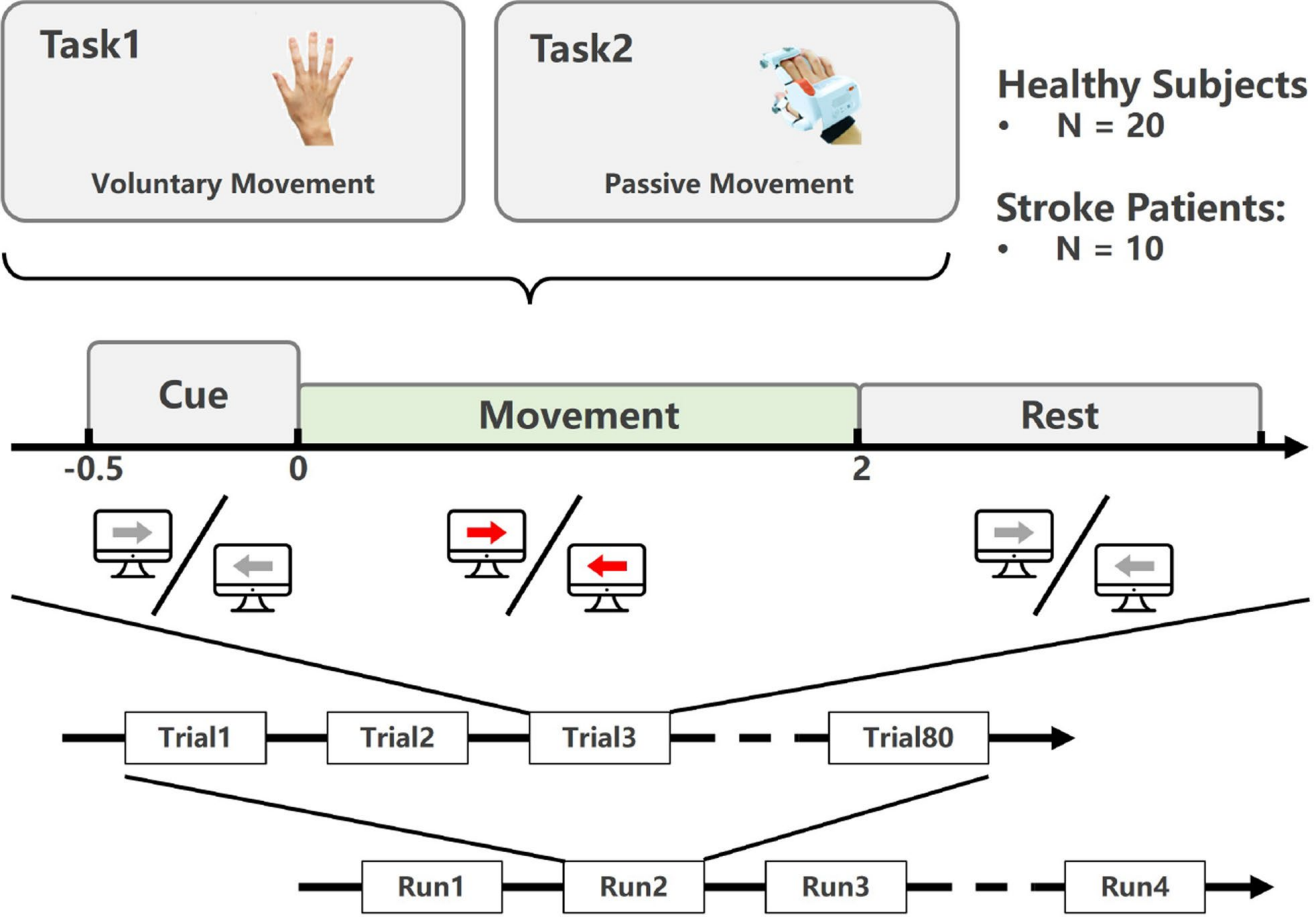
Experimental paradigm and task design. This study investigated two tasks: Task1 (Voluntary Movement) and Task2 (Passive Movement). A total of 30 participants, including 20 healthy subjects and 10 stroke patients, were recruited for the experiment. The experiment consisted of four runs, each containing 80 trials (40 trials per hand). Each trial consisted of a 0.5-s cue phase, a 2-s movement phase, and a 3-s rest phase. The red arrow cue indicated the onset of the movement phase, during which participants executed the corresponding hand movement task (actual movement, or exoskeleton-assisted movement) until the cue disappeared

The experimental paradigm is illustrated in Fig. 1. During the experiment, participants were seated in comfortable chairs facing a computer screen placed at a distance of 1 meter. They were instructed to perform various types of movement tasks in response to visual cues displayed on the screen. Each visual cue was presented 0.5 s before the onset of the movement task. Upon seeing the red arrow cue, participants were required to execute the corresponding hand movement task for a duration of 2 s until the cue disappeared. Following each movement task, there was a rest interval of 3 s. No feedback was provided to the participants during the online recording sessions. All experimental paradigms were programmed and implemented using Matlab 2022a. The experiment consisted of two tasks:

- Task1 (Voluntary Movement): Participants were asked to perform the corresponding hand movement following the red arrow cue. However, in this task, real executed grasping movements were performed instead of imagery movements.
- Task2 (Passive Movement): Participants were instructed to remain relaxed and avoid actively exerting force or participating in motion control but rather to passively focus on the sensory feedback induced by the exoskeleton movement. Synchronized with the red arrow cue, the left or right hand exoskeleton would perform either a hand-opening or hand-closing movement during the 2-s movement period.

The experiment consisted of four runs, with each task repeated twice. Each run comprised 80 randomized movement trials, evenly divided between the left and right hands (40 trials each). The study recruited 30 participants, including 20 healthy individuals and 10 stroke patients. Participants were allowed to rest for as long as they desired between consecutive runs.

During the experiment, EEG signals were acquired using a 64-channel EEG electrode system (Easycap) in combination with a BrainAmp amplifier (Brain Products GmbH, Germany). The electrodes were positioned according to the standard 10–20 system, with FCz serving as the reference channel. The EEG signals were sampled at a rate of 5000 Hz. The contact impedance between each EEG electrode and the participant’s scalp was carefully monitored and maintained below 20 $k\Omega$ prior to data acquisition. The eCon-Hand exoskeleton device (Shanghai Niantong Intelligent Co. Ltd., Shanghai, China) were used to provide full assistance for passive hand movements. The device offers a range of motion of 38 degrees for the metacarpophalangeal joint and 18 degrees for the proximal interphalangeal joint, with a maximum assistive force of 15 N. MATLAB established a wireless Bluetooth connection to synchronize with the exoskeleton device while simultaneously sending triggers to a BrainAmp amplifier via the wired parallel port.

### Data processing

#### Signal pre-processing

The raw EEG data underwent several pre-processing steps. First, the data were re-referenced to TP9 and TP10. Next, bad channel interpolation was performed to address any problematic channels. Independent Component Analysis (ICA) was then applied to remove artifacts from the data [23]. The signal was filtered using fourth-order Butterworth filters, with bandpass cutoff frequencies set to 0.01–10 Hz for time-domain analysis and 0.5–40 Hz for time-frequency analysis. Subsequently, the signal was downsampled to 200 Hz and segmented from − 3 to 6 s relative to the event of interest.

To simplify the analysis, the topographies for left-hand movements were flipped and merged with right-hand movements, resulting in an additional 40 trials for each run. In the subsequent analysis, the focus was on the neural oscillations in the motor cortices ipsilateral (Ci) and contralateral (Cc) to the movement, instead of using the traditional C4 and C3 nomenclature.

#### Time-domain analysis

For time-domain analysis, trials corresponding to the same condition were averaged. Baseline correction was then performed using a time window from − 2000 to 0 ms.

#### Time-frequency domain analysis

In the time-frequency domain analysis, a continuous wavelet transform (CWT) was applied using a complex Morlet wavelet with a central frequency of 1 Hz and a bandwidth parameter of 1.5 [24]. After trial averaging, baseline correction was performed by subtracting the mean dB power in the − 2500 to − 500 ms reference window [25], which corresponds to division in linear space due to the logarithmic transformation of decibel scaling.

It should be noted that, the baseline correction (− 2500 to − 500 ms) in time-frequency domain are different from that in time domain analysis (− 2000 to 0 ms).This adjustment addresses the temporal smearing inherent to continuous wavelet transform (CWT) analysis. The 0.5 s pre-stimulus buffer allows reliable estimation of baseline power spectral density while maintaining temporal alignment with behavioral events. This approach follows Cohen’s [26] recommendations for CWT normalization in EEG analysis [27].

#### Statistical analysis

The statistical analysis involved comparing two aspects: the mean amplitude of the time-domain signal from the interval of 0.4 s to 1.9 s (defined as the slow wave), and the mean power from 6 regions of interest (ROIs) in the time-frequency domain analysis (defined in Table 2). While guided by conventional definitions, our temporal-frequency parameter selection followed a data-driven approach based on three principles: (1) Avoiding circular analysis by predefining ROIs prior to statistical testing, (2) Balancing physiological plausibility with experimental observations through an iterative consensus process involving both engineers and clinicians, and (3) Minimizing overfitting risks through simplified boundaries (single decimal precision). These comparisons were conducted between the ipsilateral (Ci) and contralateral (Cc) sides of hand movement in healthy subjects, as well as between the unaffected and affected sides in stroke patients.

**Table 2:** Six regions of interest (ROIs) were operationalized in the time-frequency domain, with specific temporal windows designated for movement execution (ME) and movement Offset (MO) phases

ID	ROI	Frequency window (Hz)	Time window
#1	Theta_ME	3–7	0.1–0.5 s
#2	Theta_MO	3–7	2–2.5 s
#3	Mu_ME	8–13	0–2 s
#4	Mu_MO	8–13	3.2–4.2 s
#5	Beta_ME	15–28	0.5–2 s
#6	Beta_MO	15–28	2.6–3.6 s for Task 1 2.1–3.1 s for Task 2

Take ROI#1 (Theta_ME 0.1–0.5 s) for example, the time window was selected based on our data analysis, which revealed peak theta synchronization centered at 0.3 s post-trigger. To ensure symmetry around this peak, the window was defined from 0.1 to 0.5 s. Regarding the frequency band selection, the conventional theta range of 4–7 Hz was extended to 3–7 Hz based on spectral centroid analysis, which identified a characteristic peak at 4 Hz. This adjustment accounted for a 1 Hz downward shift to better capture the neurophysiological signatures of our subjects and to minimize edge-frequency bias. The mu rhythm (8–13 Hz) retained its standard definition due to its well-established association with sensorimotor rhythms. For the beta band, typically defined as 13–30 Hz, the lower bound was raised to 15 Hz to exclude mu-harmonic artifacts observed around the 13–15 Hz range in the FFT spectra. The upper limit was adjusted to 28 Hz, consistent with our wavelet analysis, which indicated that movement-related activity above this threshold was negligible, except for voluntary movements from the affected side of stroke patients.

Paired two-sample *t*-tests were employed for these comparisons. In total, there were 102 statistical comparisons. To avoid the multiple comparisons problem, the False Discovery Rate (FDR) with the Benjamini–Hochberg (BH) method was applied. The FDR-corrected *q*-value instead of the original *p*-value would be reported, with a threshold of $\alpha =0.05$.

### Machine learning

In this study, we compare the generalization ability of BCI models using two classic algorithms: CSP and EEGNet. These algorithms were chosen due to their proven effectiveness in various EEG signal processing and BCI applications.

CSP is a supervised spatial filtering technique that aims to find a set of spatial filters that maximize the variance ratio between two classes of EEG signals [28]. It has been widely used in the field due to its ability to find optimal spatial filters that maximize the discriminability between two classes of EEG signals. For the CSP method, we first applied a bandpass filter of 0.01–100 Hz to all raw EEG data to remove low-frequency drifts and high-frequency noise. An additional bandpass filter of 8–30 Hz was applied to focus on the sensorymotor rhythm (SMR) in the mu and beta bands [29], which are known to be modulated by motor imagery tasks. We then extracted a 2-s window of data starting from the onset of the task and downsampled it to 200 Hz. A total of 21 EEG channels (F3, F4, C3, C4, P3, P4, Fz, Cz, Pz, F1, F2, C1, C2, P1, P2, F5, F6, C5, C6, P5, P6) was selected for training and decoding the CSP model. Specifically, we used 2 CSP filters and the log-transformation normalized band power from 8–30 Hz as features, with LDA as the subsequent classifier. The details of this method can be found in Ramoser et al. [28].

EEGNet, proposed by Lawhern et al. [30], is a compact convolutional neural network architecture designed specifically for EEG signal analysis. By leveraging convolutional neural networks, EEGNet can automatically learn relevant features from raw EEG data, reducing the need for manual feature engineering. This makes it a promising tool for a wide range of BCI applications, as it can potentially uncover complex patterns in the data that may not be easily identified by traditional methods. For the EEGNet approach, after applying a 0.01–100 Hz bandpass filter on raw EEG data, we extracted a window of data from 0.5 s before the task cue onset to 3 s after the task end (− 0.5 s to 3 s) and downsampled it from 1000 to 200 Hz. Unlike the traditional CSP method that relies on time, spatial, and frequency domain features, EEGNet uses almost raw EEG signals as input. This type of end-to-end model allows the model to access more comprehensive information and potentially capture complex patterns using deep learning techniques. However, this approach also introduces higher complexity and dimensionality to the model, which can lead to longer training times, increased risk of overfitting, and a greater need for regularization.

To evaluate the performance of these two methods, we conducted both within-subject and cross-subject decoding analyses. For the within-subject analysis, we performed a tenfold cross-validation on each subject’s data under each paradigm. Specifically, we randomly selected 90% of a subject’s data in a paradigm for training and used the remaining 10% for testing, repeating this process ten times. The final accuracy was calculated as the average of the ten experimental runs.

For the cross-subject decoding analysis, we performed a tenfold cross-validation on all subjects’ data within each paradigm, separately for healthy subjects and stroke patients. In the healthy subject group, we randomly selected data from 18 subjects as the training set and used the remaining 2 subjects’ data as the test set, repeating this process ten times. The final accuracy was calculated as the average of the ten experimental runs. For the stroke patient group, we randomly selected data from 9 subjects as the training set and used the remaining subject’s data as the test set.

By comparing the performance of CSP and EEGNet in both within-subject and cross-subject decoding analyses, we aim to provide insights into the generalization ability of these two algorithms in various BCI applications, as well as their potential for use in clinical settings, such as stroke rehabilitation.

## Result

**Fig. 2:**
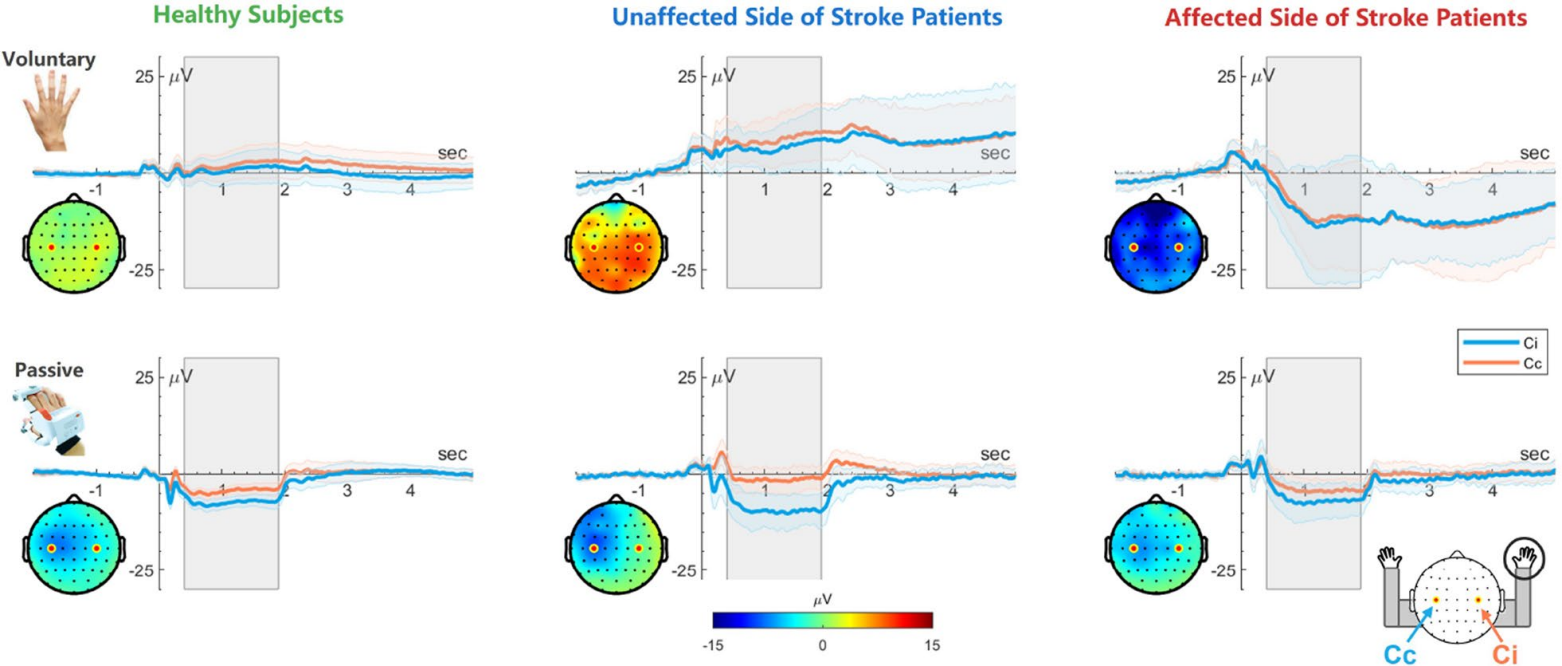
The time domain response with 95% confidence interval for the mean was compared between healthy subjects (N = 20) and stroke patients(N = 10), considering both the unaffected and affected sides. The topographies for the time window of 0.4 to 1.9 s are illustrated for each condition

**Fig. 3:**
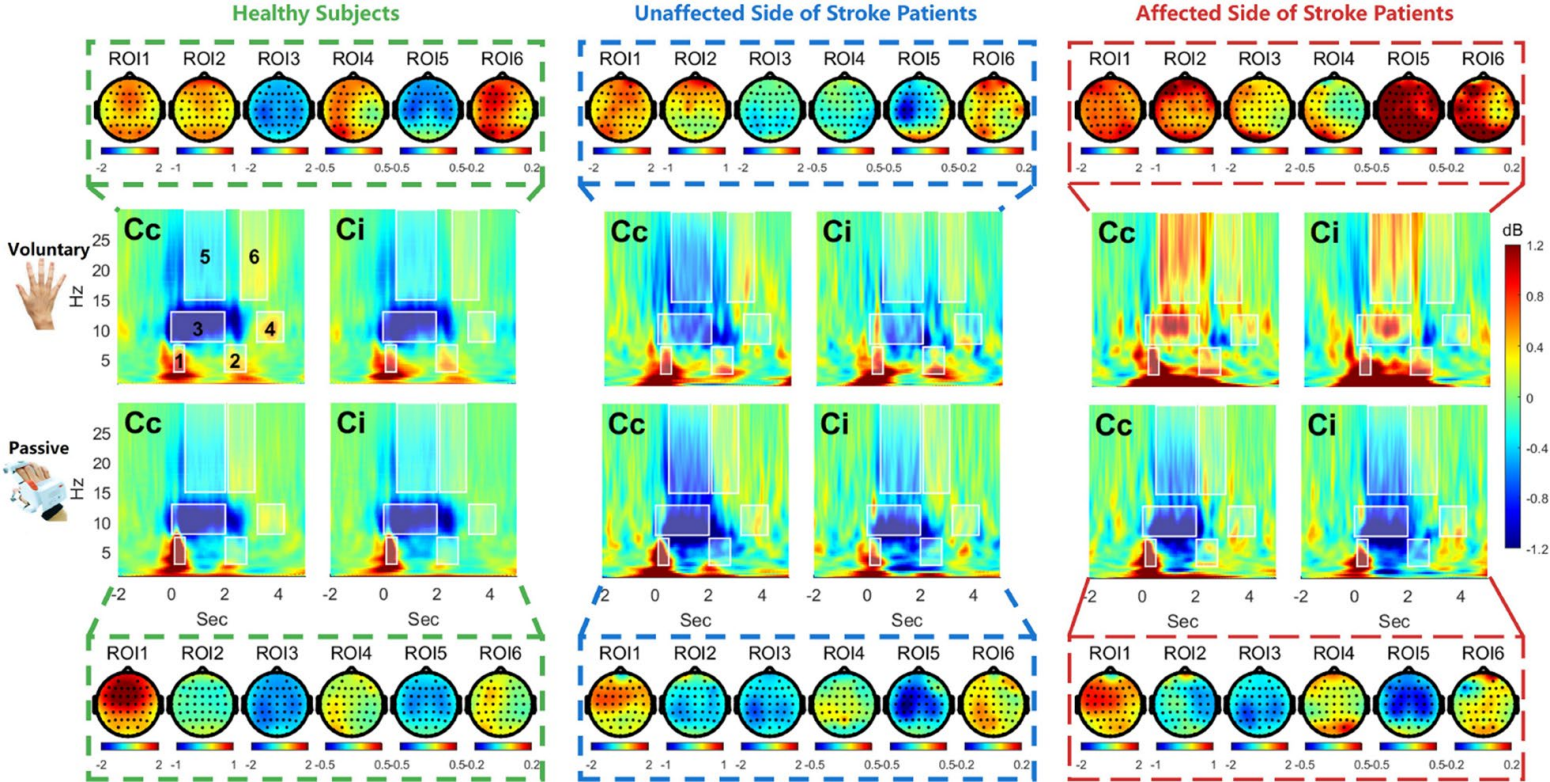
The time-frequency domain response was compared between healthy subjects and stroke patients, considering both the unaffected and affected sides. For each condition, the topographies of the six ROIs are also illustrated to provide a comprehensive spatial representation of the neural activity patterns

In the following sections, the grand averaging results for the time domain and time-frequency domain are presented in Figs. 2 and 3, respectively. The corresponding statistical analysis results for channels Cc and Ci are shown in Table 3, while those for channel FCz are presented in Table 4. To assess the generalization ability, all within-subject and cross-subject results obtained using CSP and EEGNet are illustrated in Table 5, providing a comprehensive overview of the performance of these two methods across different experimental conditions and subject groups.

**Table 3:** Statistical analyses were conducted on the time domain and time-frequency domain results for channels Cc, Ci, and the difference between channels CC and Ci (CC-Ci)

	Cc		Ci		Cc-Ci	
	Mean ± std	*q*-value	Mean ± std	*q*-value	Mean ± std	*q*-value
HS						
VOL						
Slow Wave	0.87 ± 11.50	0.73	2.03 ± 11.63	0.45	− 1.16 ± 3.19	0.07
Mu_ME(#3)	**− 1.22** ± **1.38**	**1.64E−05**	**− 1.03** ± **1.20**	**2.56E−05**	− 0.19 ± 0.72	0.20
Mu_MO(#4)	**0.29** ± **0.49**	**4.51E−03**	0.00 ± 0.33	0.97	**0.28** ± **0.51**	**5.85E−03**
Beta_ME(#5)	**− 0.32** ± **0.35**	**1.59E−05**	**− 0.28** ± **0.30**	**6.43E−06**	− 0.03 ± 0.17	0.42
Beta_MO(#6)	**0.14** ± **0.25**	**4.54E−03**	0.05 ± 0.23	0.30	**0.09** ± **0.21**	**0.03**
PAS						
Slow Wave	**− 7.29** ± **4.51**	**1.41E−10**	**− 4.33** ± **3.69**	**1.20E−07**	**− 2.96** ± **2.29**	**2.84E−08**
Mu_ME(#3)	**− 1.14** ± **0.97**	**1.20E−07**	**− 1.08** ± **0.98**	**3.60E−07**	− 0.06 ± 0.61	0.68
Mu_MO(#4)	0.15 ± 0.39	0.06	− 0.01 ±.33	0.89	**0.15** ± **0.35**	**0.03**
Beta_ME(#5)	**− 0.25** ± **0.27**	**6.27E−06**	**− 0.24** ± **0.21**	**1.46E−07**	− 0.01 ± 0.14	0.71
Beta_MO(#6)	0.06 ± 0.21	0.16	− 0.01 ± 0.14	0.73	**0.07** ± **0.15**	**0.02**
USP						
VOL						
Slow Wave	6.73 ± 7.78	0.06	**8.72** ± **8.39**	**0.03**	− 1.99 ± 4.60	0.35
Mu_ME(#3)	− 0.57 ± 0.94	0.17	− 0.42 ± 0.71	0.17	− 0.15 ± 0.46	0.50
Mu_MO(#4)	− 0.07 ± 0.41	0.70	− 0.10 ± 0.37	0.60	0.03 ± 0.48	0.89
Beta_ME(#5)	− 0.47 ± 0.56	0.07	− 0.15 ± 0.45	0.50	**− 0.32** ± **0.31**	**0.03**
Beta_MO(#6)	0.07 ± 0.33	0.68	0.03 ± 0.26	0.80	0.04 ± 0.48	0.81
PAS						
Slow Wave	**− 9.17** ± **5.84**	**4.54E−03**	− 1.35 ± 4.21	0.51	**− 7.83** ± **4.88**	**4.51E−03**
Mu_ME(#3)	− 1.20 ± 1.67	0.12	− 0.87 ± 1.27	0.13	− 0.33 ± 0.56	0.17
Mu_MO(#4)	0.10 ± 0.38	0.60	0.03 ± 0.29	0.82	0.07 ± 0.35	0.68
Beta_ME(#5)	**− 0.51** ± **0.35**	**6.22E−03**	**− 0.32** ± **0.25**	**0.01**	− 0.19 ± 0.30	0.16
Beta_MO(#6)	**0.09** ± **0.09**	**0.03**	0.02 ± 0.10	0.70	0.07 ± 0.09	0.07
ASP						
VOL						
Slow Wave	− 10.43 ± 18.00	0.19	− 9.01 ± 14.35	0.16	− 1.43 ± 7.53	0.70
Mu_ME(#3)	0.47 ± 2.34	0.68	0.26 ± 2.11	0.80	0.22 ± 0.84	0.60
Mu_MO(#4)	0.12 ± 0.50	0.64	− 0.06 ± 0.43	0.76	0.18 ± 0.46	0.41
Beta_ME(#5)	0.48 ± 1.49	0.50	0.32 ± 1.52	0.68	0.16 ± 0.49	0.50
Beta_MO(#6)	0.16 ± 0.24	0.13	0.03 ± 0.19	0.71	0.13 ± 0.20	0.15
PAS						
Slow Wave	**− 6.48** ± **5.89**	**0.03**	− 3.81 ± 4.14	0.06	− 2.67 ± 5.43	0.27
Mu_ME(#3)	− 1.11 ± 1.34	0.07	− 1.04 ± 1.51	0.13	− 0.07 ± 0.66	0.81
Mu_MO(#4)	0.04 ± 0.41	0.81	0.04 ± 0.48	0.83	0.00 ± 0.22	1.00
Beta_ME(#5)	**− 0.37** ± **0.37**	**0.03**	**− 0.40** ± **0.34**	**0.02**	0.03 ± 0.29	0.81
Beta_MO(#6)	0.04 ± 0.16	0.64	0.04 ± 0.16	0.60	0.00 ± 0.12	0.94

The analyses included data from three groups: healthy subjects (HS), the unaffected side of stroke patients (USP), and the affected side of stroke patients (ASP). Additionally, two movement conditions were considered: voluntary movement (VOL) and passive movement (PAS). The comprehensive statistical analysis aimed to uncover potential differences in neural activity patterns across channels, subject groups, and movement types. Slow wave refers to the low-frequency, time-locked ERP response

**Table 4:** Statistical analyses were performed on the time-frequency domain results obtained from channel FCz in theta band

	FCz	
	Mean ± std	*q*-value
HS		
VAL		
Theta_ME(#1)	**1.09** ± **1.17**	**7.25E−06**
Theta_MO(#2)	**0.51** ± **0.81**	**2.27E−03**
PAS		
Theta_ME(#1)	**2.10** ± **1.68**	**4.22E−08**
Theta_MO(#2)	− 0.09 ± 0.65	0.53
USP		
VAL		
Theta_ME(#1)	**1.02** ± **0.72**	**7.77E−03**
Theta_MO(#2)	0.34 ± 0.49	0.13
PAS		
Theta_ME(#1)	**1.06** ± **0.86**	**0.02**
Theta_MO(#2)	−0.26 ± 1.01	0.61
ASP		
VAL		
Theta_ME(#1)	**1.05** ± **0.78**	**9.64E−03**
Theta_MO(#2)	0.51 ± 0.93	0.22
PAS		
Theta_ME(#1)	**1.29** ± **1.10**	**0.02**
Theta_MO(#2)	− 0.27 ± 0.73	0.44

The analyses included data from three groups: healthy subjects (HS), the unaffected side of stroke patients (USP), and the affected side of stroke patients (ASP). Additionally, two movement conditions were considered: voluntary movement (VOL) and passive movement (PAS). The comprehensive statistical analysis aimed to uncover potential differences in neural activity patterns across channels, subject groups, and movement types

**Table 5:** Generalization ability analysis results for voluntary and passive movements using CSP and EEGNet classifiers

	Voluntary		Passive	
	CSP	EEGNET	CSP	EEGNET
Within subject				
HS	60.81	58.38	62.66	62.47
SP	81.25	84.19	67.62	80.13
Cross subject				
HS	56.28	64.75	58.91	86.00
SP	54.94	67.81	55.81	72.63

The within-subject and cross-subject accuracies (%) are presented for healthy subjects (HS) and stroke patients (SP)

### Time domain analysis

The grand-averaged event-related potentials (ERPs) for voluntary and passive movements in healthy subjects, as well as in the unaffected and affected sides of stroke patients, are illustrated in Fig. 2. The ERPs are shown for both the contralateral (Cc) and ipsilateral (Ci) channels. Additionally, the corresponding topographies representing the mean amplitude during the 0.4–1.9 s after movement onset are plotted alongside the ERPs.

#### Voluntary and passive movements in healthy subjects

The average amplitude of cortical potentials during the 0.4–1.9 s interval after movement onset was evaluated in healthy subjects (Fig. 2, first column). Healthy subjects exhibited significant negative cortical potentials during passive movements, while no significant changes were observed during voluntary movements. For voluntary movements, the decreases in cortical potentials was not significant for either the contralateral (Cc: $0.87\pm 11.50$ μV, FDR corrected $q=0.73$) or ipsilateral (Ci: $2.03\pm 11.63$ μV, FDR corrected $q=0.45$) sides. In contrast, during passive movements, significant decreases in cortical potentials were observed for both the contralateral (Cc: $-7.29 \pm 4.51$ μV, FDR-corrected $q=1.41 \times 10^{-10}$) and ipsilateral (Ci: $-4.33 \pm 3.69$ μV, FDR-corrected $q=1.20 \times 10^{-7}$) sides.

A lateralization effect was observed, with contralateral cortical potentials consistently lower than ipsilateral potentials, regardless of the movement type. This lateralization effect was more pronounced during passive movements (Cc-Ci difference: voluntary $-1.16\pm 3.19$ μV, FDR corrected $q=0.07$; passive $-2.96\pm 2.29$ μV, FDR corrected $q=5.57 \times 10^{-8}$).

#### Voluntary movements in stroke patients

Stroke patients exhibited different patterns of cortical potential changes during voluntary movements of the unaffected and affected sides compared to healthy subjects. For voluntary movements of the unaffected side, stroke patients showed a marginal increase in theta-band oscillations in both hemispheres (Cc: $6.73\pm 7.78~\upmu V$, FDR corrected $q=0.06$; Ci: $8.72\pm 8.39~\upmu V$, FDR corrected $q=0.03$). In contrast, during voluntary movements of the affected side, the decrease in cortical potentials was larger for both contralateral and ipsilateral sides but did not reach statistical significance (Cc: $-10.43\pm 18.00~\upmu V$, FDR corrected $q=0.19$; Ci: $-9.01\pm 14.35~\upmu V$, FDR corrected $q=0.16$).

The lateralization effect was not evident during voluntary movements of either the unaffected (Cc-Ci difference: $-1.99\pm 4.60~\upmu V$, FDR corrected $q=0.35$) or affected (Cc-Ci difference: $-1.43\pm 7.53~\upmu V$, FDR corrected $q=0.70$) side. Furthermore, after the movement ended, the cortical potentials did not return to baseline levels for an extended period for both the unaffected and affected side movements.

#### Passive movements in stroke patients

Passive movements in stroke patients resulted in a decrease in cortical potentials for both the unaffected (Cc: $-9.17\pm 5.84~\upmu V$, FDR corrected $q=4.54 \times 10^{-3}$; Ci: $-1.35\pm 4.21~\upmu V$, FDR corrected $q=0.51$) and affected (Cc: $-6.48\pm 5.89~\upmu V$, FDR corrected $q=0.03$; Ci: $-3.81\pm 4.14~\upmu V$, FDR corrected $q=0.06$) sides.

A significant lateralization effect was found during passive movements of the unaffected side (Cc-Ci difference: $-7.83\pm 4.88~\upmu V$, FDR corrected $q=4.51 \times 10^{-3}$), but this was not found on the affected side (Cc-Ci difference: $-2.67\pm 5.43~\upmu V$, FDR corrected $q=0.27$). Unlike voluntary movements, the cortical potentials returned to baseline levels immediately after the passive movement ended.

Notably, the pattern of cortical potential changes during passive movements in stroke patients, for both the unaffected and affected sides, more closely resembled that of healthy subjects compared to the pattern observed during voluntary movements. More importantly, the inter-individual consistency was significantly better during exoskeleton-guided passive movements compared to active movements. The variance of slow wave potentials was much smaller, stabilizing between 3.5 and 6, regardless of whether it was in healthy subjects, the unaffected side of patients, or the affected side of patients, and regardless of whether it was the ipsilateral or contralateral brain under different conditions.

### Time-frequency domain analysis

#### Voluntary and passive movements in healthy subjects

Firstly, we compared the brain activity during voluntary and passive movements to understand the neural mechanisms underlying motor control and sensory feedback processing in healthy subjects (Fig. 3, first column). By analyzing the differences in EEG responses between these two conditions, we can gain valuable insights into how the brain coordinates and perceives movements. In this section, we investigate the variations in EEG responses of healthy subjects during voluntary and passive movements, focusing on three key frequency bands: theta (3–7 Hz), mu (8–13 Hz), and beta (15–28 Hz) rhythms. We examine the spatiotemporal characteristics of these rhythms, including ERD and ERS, and assess the lateralization effects between the contralateral and ipsilateral motor cortices.

- Theta rhythm analysis: For the theta rhythm (3–7 Hz), two ROIs were defined based on movement onset and offset: ROI#1 (0.1–0.5 s) and ROI#2 (2–2.2– s). The most pronounced activity was observed not in the sensorimotor area (Cc or Ci channel), but in the frontal lobe, specifically at the FCz channel. At movement onset in ROI#1, both voluntary and passive movements showed a significant increase in theta power, mainly localized in the prefrontal area rather than the motor regions Cc and Ci. At the FCz channel, passive movement ($2.10 \pm 1.68$ dB, FDR corrected $q=4.22\times 10^{-8}$) exhibited higher power than voluntary movement ($1.09 \pm 1.17$ dB, FDR corrected $q=7.25 \times 10^{-6}$). In contrast, at movement offset in ROI#2, the FCz channel showed a power decrease in passive movement ($-0.09 \pm 0.65$ dB, FDR corrected $q=0.53$) compared to voluntary movement ($0.51 \pm 0.81$ dB, FDR corrected $q=2.27 \times 10^{-3}$).
- Mu rhythm analysis: For the mu rhythm (8–13 Hz), two ROIs were defined: ROI#3 (0–2 s) and ROI#4 (3.2–4 s). During movement execution in ROI#3, both voluntary (Cc, $-1.22\pm 1.38$ dB, FDR corrected $q=1.64\times 10^{-5}$; Ci, $-1.03\pm 1.20$ dB, FDR corrected $q=2.56\times 10^{-5}$) and passive movements (Cc, $-1.14\pm 0.97$ dB, FDR corrected $q=1.20\times 10^{-7}$; Ci, $-1.08\pm 0.98$ dB, FDR corrected $q=3.60\times 10^{-7}$) showed significant ERD. Lateralization was not evident in either voluntary (Cc-Ci, $-0.19\pm 0.72$ dB, FDR corrected $q=0.20$) or passive movement (Cc-Ci, $-0.06\pm 0.61$ dB, FDR corrected $q=0.68$). After movement offset in ROI#4, contralateral side ERS was observed significantly for voluntary movement (Cc, $0.29\pm 0.49$ dB, FDR corrected $q=4.51\times 10^{-3}$), but marginal for passive movements (Cc, $0.15\pm 0.39$ dB, FDR corrected $q=0.06$). The ipsilateral channel did not show significant ERS in either voluntary (Ci, $0.00\pm 0.33$ dB, FDR corrected $q=0.97$) or passive movement (Ci, $-0.01\pm 0.33$ dB, FDR corrected $q=0.89$). Therefore, significant lateralization was present in both voluntary (Cc-Ci, $0.28\pm 0.51$ dB, FDR corrected $q=5.85\times 10^{-3}$) and passive movements (Cc-Ci, $0.15\pm 0.35$ dB, FDR corrected $q=0.03$).
- Beta rhythm analysis: For the beta rhythm (15–28 Hz), two ROIs were defined: ROI#5 (0.5–2 s) and ROI#6 (voluntary: 2.6–3.6 s; passive: 2.1–3.1 s). The statistical results are similar to those of the mu rhythm analysis. During movement execution in ROI#5, both voluntary (Cc, $-0.32\pm 0.35$ dB, FDR corrected $q=1.59\times 10^{-5}$; Ci, $-0.28\pm 0.30$ dB, FDR corrected $q=6.43\times 10^{-6}$) and passive movements (Cc, $-0.25\pm 0.27$ dB, FDR corrected $q=6.27\times 10^{-6}$; Ci, $-0.24\pm 0.21$ dB, FDR corrected $q=1.46\times 10^{-7}$) showed significant ERD. Lateralization was not evident in either voluntary (Cc-Ci, $-0.03\pm 0.17$ dB, FDR corrected $q=0.42$) or passive movement (Cc-Ci, $-0.01\pm 0.14$ dB, FDR corrected $q=0.71$). After movement offset in ROI#6, only the contralateral channel in voluntary movement (Cc, $0.14\pm 0.27$ dB, FDR corrected $q=4.54\times 10^{-3}$) exhibited significant ERS. The ipsilateral Ci channel in voluntary movement (Ci, $0.05\pm 0.23$ dB, FDR corrected $q=0.30$) and both channels in passive movement (Cc, $0.06\pm 0.21$ dB, FDR corrected $q=0.16$; Ci, $-0.01\pm 0.14$ dB, FDR corrected $q=0.73$) did not show significant ERS. Significant lateralization was present in both voluntary (Cc-Ci, $-0.09\pm 0.21$ dB, FDR corrected $q=0.03$) and passive movements (Cc-Ci, $0.07\pm 0.15$ dB, FDR corrected $q=0.02$). The beta rhythm ERD in ROI#6 occurred earlier in passive movement compared to the mu rhythm ERD in ROI#4, with a difference of approximately 0.5 s.

#### Voluntary movements in stroke patients

In stroke patients, brain activity patterns during voluntary movements differ between frequency bands when compared to healthy subjects. Theta band activity remains similar for both the unaffected and affected sides, showing no significant differences in power or spatial distribution. However, mu and beta bands exhibit distinct patterns between the unaffected and affected sides, deviating from those observed in healthy individuals.

- Theta rhythm: The theta rhythm power induced by voluntary movements in stroke patients is similar to that in healthy subjects. In ROI#1, both the unaffected side (USP) ($1.02\pm 0.72$ dB, FDR corrected $q=7.77\times 10^{-3}$) and the affected side (ASP) ($1.05\pm 0.78$ dB, FDR corrected $q=9.64\times 10^{-3}$) show significant power increases at the FCz channel. Similarly, in ROI#2, the theta power remains elevated, although not statistically significant (USP: $0.34\pm 0.49$ dB, FDR corrected $q=0.13$; ASP: $0.51\pm 0.93$ dB, FDR corrected $q=0.22$). The topographic maps show a more dispersed distribution of theta activity in stroke patients rather than being concentrated at FCz, as observed in healthy subjects.
- Mu rhythm: Significant differences are observed between the unaffected and affected sides in stroke patients’ voluntary movement mu rhythm. In ROI#3, the unaffected side movement shows reduced power changes (Cc: $-0.57\pm 0.94$ dB, FDR corrected $q=0.17$; Ci: $-0.42\pm 0.71$ dB, FDR corrected $q=0.17$) with a smaller magnitude compared to healthy subjects, while the affected side movement shows positive power changes (Cc: $0.47\pm 2.34$ dB, FDR corrected $q=0.68$; Ci: $0.26\pm 2.11$ dB, FDR corrected $q=0.80$), although neither reaches statistical significance. After movement offset in ROI#4, the trending toward ERS-like patterns is not significant for either side (unaffected side: Cc, $-0.07\pm 0.41$ dB, FDR corrected $q=0.70$; Ci, $-0.10\pm 0.37$ dB, FDR corrected $q=0.60$; affected side: Cc, $0.12\pm 0.50$ dB, FDR corrected $q=0.64$; Ci, $-0.06\pm 0.43$ dB, FDR corrected $q=0.76$). Interestingly, a brief ERD is observed in the interval between ROI#3 and ROI#4 ($<span class='convertEndash'>2.5-3</span>$ s) for both unaffected and affected side movements, occurring slightly later than in healthy subjects.
- Beta rhythm: Similar to the mu rhythm, significant differences are observed between the unaffected and affected sides in stroke patients’ voluntary movement beta rhythm. In ROI#5, the unaffected side movement shows the trending toward ERD-like patterns (Cc: $-0.47\pm 0.56$ dB, FDR corrected $q=0.07$; Ci: $-0.15\pm 0.45$ dB, FDR corrected $q=0.50$), with a more pronounced contralateral effect compared to healthy subjects (Cc-Ci: $-0.32\pm 0.31$ dB, FDR corrected $q=0.03$). The affected side movement shows positive power changes (Cc: $0.48\pm 1.49$ dB, FDR corrected $q=0.50$; Ci: $0.32\pm 1.52$ dB, FDR corrected $q=0.68$), but neither reaches statistical significance, and the contralateral effect is also not significant ($0.16\pm 0.49$ dB, FDR corrected $q=0.50$). After movement offset in ROI#6, positive power changes is not significant for either side (unaffected side: Cc, $0.07\pm 0.33$ dB, FDR corrected $q=0.68$; Ci, $0.03\pm 0.26$ dB, FDR corrected $q=0.80$; affected side: Cc, $0.48\pm 1.49$ dB, FDR corrected $q=0.50$; Ci, $0.32\pm 1.52$ dB, FDR corrected $q=0.68$).

#### Passive movements in stroke patients

EEG signals during passive movement in stroke patients were more consistent with those of healthy subjects compared to patients’ voluntary movement. The results for theta, mu, and beta rhythms are as follows:

- Theta rhythm: In ROI#1, both the unaffected side (USP) ($1.06\pm 0.86$ dB, FDR corrected $q=0.02$) and affected side (ASP) ($1.29\pm 1.10$ dB, FDR corrected $q=0.02$) showed significant power increases at the FCz channel. In ROI#2, the theta power remained elevated but not statistically significant (USP: $-0.26\pm 1.01$ dB, FDR corrected $q=0.61$; ASP: $-0.27\pm 0.73$ dB, FDR corrected $q=0.44$). The topographic maps showed a very similar distribution to healthy subjects.
- Mu rhythm: In ROI#3, the reduced power changes during unaffected side movement (Cc: $-1.20\pm 1.67$ dB, FDR corrected $q=0.12$; Ci: $-0.87\pm 1.27$ dB, FDR corrected $q=0.13$) and affected side movement (Cc: $-1.11\pm 1.34$ dB, FDR corrected $q=0.07$; Ci: $-1.04\pm 1.51$ dB, FDR corrected $q=0.13$) was very similar to passive movement in healthy subjects. However, there was greater inter-individual variability, with two patients not showing significant ERD. The peak frequency was lower than in healthy subjects on average, and the power of ERD weakened after movement offset, unlike the 0.5 s persistence seen in healthy subjects. In ROI#4, positive power changes was not significant for either unaffected side movement (Cc: $0.10\pm 0.38$ dB, FDR corrected $q=0.60$; Ci: $0.03\pm 0.29$ dB, FDR corrected $q=0.82$) or affected side movement (Cc: $0.04\pm 0.41$ dB, FDR corrected $q=0.81$; Ci: $0.04\pm 0.48$ dB, FDR corrected $q=0.83$).
- Beta rhythm: In ROI#5, patients showed significant ERD during both unaffected side movement (Cc: $-0.51\pm 0.35$ dB, FDR corrected $q=6.22\times 10^{-3}$; Ci: $-0.32\pm 0.25$ dB, FDR corrected $q=0.01$) and affected side movement (Cc: $-0.37\pm 0.37$ dB, FDR corrected $q=0.03$; Ci: $-0.40\pm 0.34$ dB, FDR corrected $q=0.02$), with good consistency observed in all subjects. Compared to passive movement in healthy subjects, the amplitude was lower, and the ERD significantly weakened after movement offset, unlike the 0.5 s persistence seen in healthy subjects. In ROI#6, although the amplitude was relatively weak and may not be clearly visible in the time-frequency plots, the corresponding topographic maps showed significant ERS in the contralateral channel during unaffected side movement (Cc: $0.09\pm 0.09$ dB, FDR corrected $q=0.03$). The other conditions, including unaffected side movement (Ci: $0.02\pm 0.10$ dB, FDR corrected $q=0.64$) and affected side movement (Cc: $0.04\pm 0.16$ dB, FDR corrected $q=0.64$; Ci: $0.04\pm 0.16$ dB, FDR corrected $q=0.60$), did not show significant ERS.

### Generalization ability analysis

Table 5 compares the performance of two BCI algorithms, CSP and EEGNet, in both within-subject and cross-subject decoding analyses for healthy subjects (HS) and stroke patients (SP) under voluntary and passive movement paradigms.

For within-subject decoding, the mean accuracy for healthy subjects is not high for both CSP and EEGNet in voluntary and passive movement paradigms. However, for stroke patients, both CSP and EEGNet achieve high accuracy in the voluntary movement paradigm, with CSP reaching 81.25% and EEGNet achieving 84.19%. This may be explained by the distinct brain activity patterns in both time and time-frequency domains between the unaffected and affected side movements. In the passive movement paradigm, the accuracies are lower for CSP at 67.62% but improve significantly with EEGNet at 80.13% for stroke patients.

In the cross-subject decoding analysis, the accuracies with CSP are lower than within-subject decoding, as expected due to the increased variability across subjects. For healthy subjects, CSP achieves 56.28% accuracy in the voluntary movement paradigm and 58.91% in the passive movement paradigm. Interestingly, EEGNet reaches higher accuracies of 64.75% and 86.00% in the voluntary and passive movement paradigms, respectively, surpassing the within-subject decoding performance. For stroke patients, CSP shows accuracies of 54.94% and 55.81% in the voluntary and passive movement paradigms, while EEGNet achieves 67.81% and 72.63%, respectively. These accuracies are not higher than the within-subject decoding results for stroke patients.

## Conclusion and discussion

In this study, we conducted a comprehensive investigation of EEG responses during voluntary and passive movements in healthy subjects and stroke patients. By employing time domain and time-frequency domain analysis and foundational traditional machine learning and deep learning algorithms, we uncovered novel insights into the neural mechanisms underlying motor control and sensory feedback processing, as well as their potential applications in BCI systems for stroke rehabilitation.

### Slow wave potential

One of the key findings of our study is that passive movements elicit more pronounced and consistent EEG responses compared to voluntary movements, particularly in the slow wave component (refers to the low-frequency, time-locked ERP response). In healthy subjects, passive movements evoked significantly stronger slow wave negative potentials bilaterally in the motor cortex, with the decrease in cortical potentials being highly significant for both the contralateral (Cc: $-7.29 \pm 4.51 \mu V$, FDR corrected $q = 1.41 \times 10^{-10}$) and ipsilateral (Ci: $-4.33 \pm 3.69 \mu V$, FDR corrected $q = 1.20 \times 10^{-7}$) sides. Importantly, baseline correction (− 2 to 0 s) was applied to account for pre-movement activity, ensuring observed differences are not confounded by preparation effects. Notably, no preparation-related negative deflections were detected in the adjusted − 0.5 to 0 s window, reinforcing the validity of these amplitude comparisons. While movement-related cortical potentials (MRCPs) are predominantly associated with volitional motor planning and execution in self-initiated movements [31–33], emerging evidence suggests distinct neural substrates for passive movements. Specifically, passive motor tasks engage proprioceptive feedback mechanisms that activate primary somatosensory cortices, generating slow wave potentials that may exhibit higher amplitude characteristics compared to voluntary movement-related cortical activity in certain experimental paradigms [34]. This amplitude discrepancy observed in our findings aligns with prior neurophysiological investigations into sensorimotor integration pathways [32, 34]. Whether the slow wave potentials evoked by passive movements share neurophysiological origins with MRCPs or reflect distinct sensorimotor integration mechanisms remains to be elucidated.

For stroke patients, the EEG response patterns in the affected and unaffected hemispheres during voluntary movement tasks differed significantly from those of healthy subjects. It should be noted that the upward trend preceding the baseline in the voluntary movement of stroke patients reflects residual signals from the preceding trial’s movement termination (recorded 3–5 s post-motion), averaged across unaffected and affected sides. We do not attribute this to preparation potentials, as the observed trend temporally misaligns with their expected time scale. Patients exhibited prolonged persistence of slow wave potentials following voluntary movements, contrasting with the more dynamic modulation observed in healthy individuals. Notably, while both groups lacked strong interhemispheric asymmetry during active movement, this phenomenon in patients likely reflects pathological mechanisms such as decreased cortical excitability, impaired interhemispheric inhibition, and compensatory overactivation of the unaffected hemisphere [35, 36]. The sustained slow wave potentials may indicate inefficient disengagement of neural resources post-movement, potentially related to increased cognitive effort for motor execution or disrupted sensorimotor integration. Interestingly, passive movements of both limbs in patients elicited response patterns more akin to active movements in healthy individuals. This observation could cautiously suggest that while stroke patients’ volitional motor execution is compromised, their sensorimotor systems retain some capacity to process proprioceptive information—a notion partially supported by the preserved tactile sensation in our cohort (though direct proprioceptive assessments were not performed). The neural pattern similarities between passive patient movements and active healthy movements may reflect either relatively intact sensory integration mechanisms or compensatory cortical reorganization, highlighting the need for future studies combining neurophysiological measures with behavioral proprioceptive evaluations [37].

In the field of BCI, decoding mainly relies on mu and beta rhythms in the time-frequency domain, while research on slow wave potentials is insufficient. Our systematic comparison of slow wave potentials between voluntary and passive movements, as well as across healthy subjects and the unaffected/affected sides in stroke patients, revealed that passive movements demonstrated better consistency across conditions and exhibited significantly reduced cross-subject variability compared to voluntary movements. This finding may account for EEGNet’s enhanced generalization capability in passive movement paradigms. Although delta band oscillations are highly informative, they are more susceptible to noise and exhibit closer proximity to the DC component, thereby requiring higher-performance amplifiers [38]. Therefore, research in this area is limited. For example, it is worth further investigating the differences in slow potential responses evoked by rigid exoskeleton mechanical hands, soft pneumatic gloves, or functional electrical stimulation.

### Cross-frequency coexistence of ERD and ERS

The dissociation between mu ERD and beta ERS reflects their distinct roles in sensorimotor integration. Mu ERD, ubiquitous in both active and passive movements, encodes real-time proprioceptive feedback, as evidenced by its correlation with joint displacement velocity during passive tasks [29, 39]. This aligns with studies showing mu rhythm desynchronization during passive limb manipulation, where sensory afference drives cortical activation without motor intent [40, 41]. Beta ERS, however, operates contextually: in active movements, it stabilizes post-execution motor networks, while in passive paradigms, it mediates sensory-driven inhibition [42, 43]. Notably, beta synchronization during passive trials-observed even in patients’ unaffected hemispheres-is not a motor stabilizer but a sensory gatekeeper. Mechanistic work [44, 45] confirms that passive beta ERS requires intact sensory pathways and suppresses spurious motor activation, a critical function in stroke patients compensating for impaired motor control. The temporal hierarchy of mu ERD (early proprioceptive processing) followed by beta ERS (later sensory-motor disengagement) underscores a dynamic coordination mechanism. This dual-phase process ensures efficient integration of kinematic feedback while preventing maladaptive motor outputs, reconciling the coexistence of these rhythms in passive trials and offering a unified model for interpreting ERD/ERS across motor states.

### Classification performance and generalization ability

In the BCI decoding aspect, we explored the performance of different machine learning algorithms in within-subject and cross-subject tasks. Notably, we demonstrated that the classification accuracy for distinguishing between affected and unaffected side movements in stroke patients was consistently higher than that for left vs. right hand movements in healthy subjects, reaching up to 84.19% with the EEGNet deep learning algorithm. This finding is particularly interesting, as most current MI-BCI studies are conducted on healthy individuals, and it is generally believed that classification performance would be lower in stroke patients [8, 11]. It is worth noting that most existing transfer learning approaches for MI-BCI have primarily utilized data from healthy subjects [17, 46, 47]. However, a recent study by Nagarajan et al. [19] highlighted the difficulty of transferring learning from healthy subjects to stroke patients and between stroke patients for voluntary movement tasks.

In our study, we showed that deep learning approaches, such as those based on EEGNet, can significantly improve cross-subject decoding performance, outperforming traditional methods like CSP + LDA. Furthermore, the higher accuracies observed in the passive movement paradigm suggest that passive movements elicit more consistent EEG response patterns across subjects, facilitating better cross-subject decoding outcomes. This may be attributed to the lower inter-subject variability (lower standard deviation as compared with voluntary movement, especially for slow wave potential). These results, along with the findings of Nagarajan et al. [19], highlight the potential for creating more robust and generalizable BCI systems that can adapt to the variability across individuals and patient populations by leveraging deep learning techniques and passive movement paradigms.

### Exoskeleton-guided movement for BCI rehabilitation

Our findings highlight the potential of incorporating exoskeleton-guided passive movement paradigms into the development of BCI systems for stroke rehabilitation. Traditional motor imagery-based BCIs have shown promise in promoting motor recovery [8, 48]; however, the generalization ability of active movement in stroke patients is often limited. In contrast, passive movements can provide better consistency and improve the performance of cross-subject decoding [49]. By engaging multiple sensory modalities and leveraging the natural neural dynamics of sensorimotor processing, passive movement-based BCIs could potentially enhance the efficacy and accessibility of stroke rehabilitation interventions [50]. However, it is important to note that the specific implementation and effectiveness of this approach in stroke rehabilitation remain to be further investigated.

**Fig. 4:**
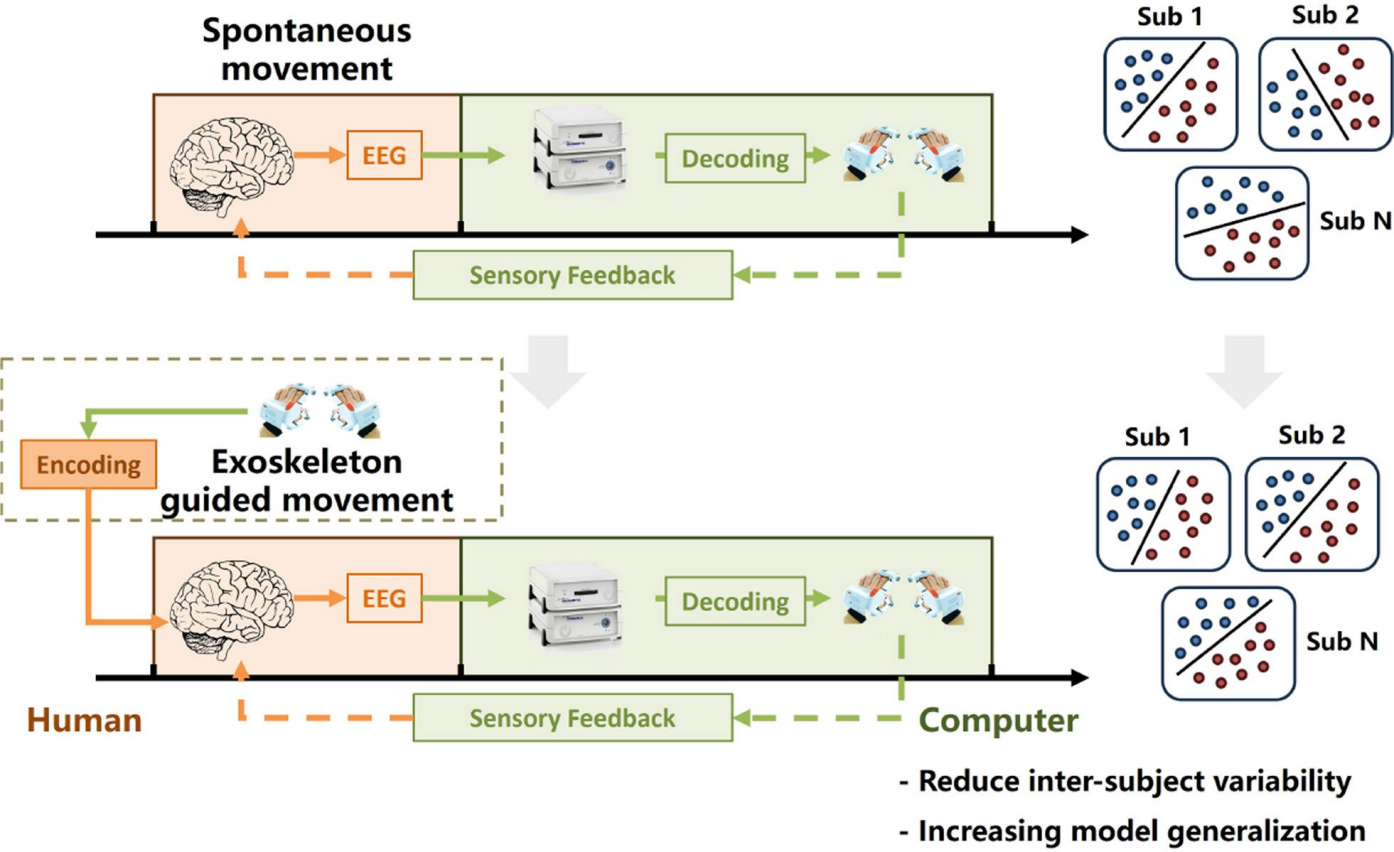
Schematic diagram of the exoskeleton-guided BCI-rehabilitation paradigm. The dashed box highlights the incorporation of external stimuli from the exoskeleton into the existing spontanous MI-BCI paradigm to reduce inter-subject variability and improve the model generalization

The inspiration is drawn from SSVEP paradigms. As a type of evoked paradigms, SSVEP utilizes external stimuli as carrier signals modulated by human subjective intention (“attention” in SSVEP), effectively improving EEG signal-to-noise ratio, reducing intra- and inter-subject variability, and even enabling training-free decoding [51] compared to spontaneous motor imagery. These advantages are urgently needed in clinical applications [52]. By incorporating external stimuli from the exoskeleton into the existing motor imagery MI-BCI paradigm (the dashed box in Fig. 4), the proposed exoskeleton-guided BCI-rehabilitation paradigm aims to reduce inter-subject variability by providing a consistent external stimulus that assists in evoking and encoding motor imagery or actual movement. This method offers a promising complementary approach to traditional voluntary motor imagery-based BCIs, which are often limited by large inter-subject and intra-subject variability [11].

We hypothesize that this closed-loop training method, targeting both the top-down motor neural pathway and the bottom-up sensory neural pathway, may be more effective than training only a single channel of the motor neural pathway. The ability to evoke consistent EEG responses across different subject groups and the improved generalization performance of deep learning algorithms highlight the feasibility of developing large-scale, standardized BCI systems that can be readily deployed in clinical settings. However, further research is needed to provide evidence on whether this exoskeleton-guided active-passive combined training method can effectively improve patients’ motor neural abilities and to address the limitations and assumptions associated with this approach.

### Conclusion

This study establishes exoskeleton-guided passive movement as a neurophysiologically standardized paradigm that addresses critical limitations in conventional BCIs for stroke rehabilitation. Through systematic comparisons of EEG signatures between voluntary and passive movements across healthy and post-stroke cohorts, we demonstrate two key advancements:

1. Standardized neural encoding: Passive movements generated significantly strong slow-wave potentials (Cc: $-7.29\upmu V$; Ci: $-4.33\upmu V$ in average), while no significant changes were observed during voluntary movements (Cc: $0.87\upmu V$; Ci: $2.03\upmu V$ in average). The standard deviation of cortical potentials during passive movements (Cc: $4.51~\upmu V$; Ci: $3.69~\upmu V$) was significantly lower than that during voluntary movements (Cc: $11.50~\upmu V$; Ci: $11.63~\upmu V$), indicating a reduction in inter-subject variability to 39% (4.51/11.50) and 32% (3.69/11.63) of that observed during voluntary movements, respectively. This neural normalization effect persisted in stroke patients, where passive movements restored physiological ERD/ERS patterns disrupted during voluntary attempts.
2. Generalizable cross-subject decoding: EEGNet achieved unprecedented cross-subject decoding accuracy (healthy: 86.00%; stroke: 72.63%) in passive paradigms, outperforming voluntary movement models by 21–28%. Exoskeleton-guided passive movement has the potential to reduce inter-subject variability and improve generalization, sparking hope for addressing the longstanding “subject calibration bottleneck” in clinical BCI implementation. However, it should be noted that passive and active movements operate under distinct neural mechanisms. As such, passive movement cannot replace active movement.

These findings would potentially reshape BCI design principles for neurological rehabilitation. By integrating objective exoskeleton-evoked responses with subjective motor imagery modulation, our approach leverages external sensory cues to reduce inter- and intra-subject variability, thereby enhancing neural response consistency and improving algorithm generalization.

In conclusion, our findings represent a significant step forward in the quest to optimize BCI technology for stroke rehabilitation. Future research should build upon these foundations, further refining passive movement paradigms, exploring their long-term effects on motor recovery, and integrating them synergistically with other neurorehabilitation techniques. Ultimately, this pioneering work paves the way for more effective, personalized, and accessible BCI systems, offering hope for improved outcomes and enhanced quality of life for stroke survivors worldwide.

Clinically, this paradigm highlights the potential of exoskeleton-guided passive movement as a training-free, ready-to-use framework (cross-subject accuracy 72.63%) for heterogeneous patient populations without individual calibration. Future work should prioritize multisite validation of long-term recovery outcomes and integration with neuromodulation techniques to synergize standardized diagnostics with personalized therapeutics.

## Supplementary Information

Supplementary Material 1

## Data Availability

The data that support the findings of this study are available from the corresponding author upon reasonable request.

## Abbreviations

BCIs: Brain-computer interfaces
EEG: Electroencephalogram
ERD: Event-related desynchronization
ERS: Event-related synchronization
MI-BCI: Motor imagery brain-computer interface
CSP: Common Spatial Patterns
HS: Healthy subjects
SP: Stroke patients
CT: Computed tomography
MRI: Magnetic resonance imaging
MMSE: Mini-Mental State Examination
ROI: Region of interest
SMR: Sensorimotor rhythm
USP: Unaffected side of stroke patients
ASP: Affected side of stroke patients
MRCP: Movement-related cortical potential
